# A Rare Case of Severe Starvation-induced Ketoacidosis in a Patient with Recurrent Pancreatitis

**DOI:** 10.7759/cureus.7368

**Published:** 2020-03-22

**Authors:** Kok Hoe Chan, Amr Ramahi

**Affiliations:** 1 Internal Medicine, Saint Michael's Medical Center, Newark, USA

**Keywords:** starvation, ketoacidosis, recurrent pancreatitis

## Abstract

Starvation-induced ketoacidosis in non-diabetic and non-pregnant, otherwise healthy patients is not common. In an otherwise normal healthy individual, short-term starving will only result in mild ketosis. Nonetheless, the effects of ketosis can become more severe if there is stress and insulin resistance, such as in pregnant or lactating woman or in very young individual such as neonates. We report a case of severe starvation-induced ketoacidosis in a non-diabetic and non-pregnant 37-year-old African American female patient with a history of multiple recurrent pancreatitis. The patient was initially presented to the emergency department with abdominal pain, nausea and vomiting over two days. The patient also reported starving for two days prior to admission. Biological findings, however, showed a severe degree of metabolic acidosis with an increased anion gap. Serum glucose was normal and 3+ ketonuria were present. Lactic acid was 1.7 mmol/L with no uremia. Salicylate acid, acetaminophen and ethanol level were normal. The patient’s beta-hydroxybutyrate level elevated with ketonuria, suggestive of ketoacidosis as the cause of metabolic acidosis. To our knowledge, the presenting case was novel as no case reports or case series have been reported in these groups of patients. Short-term starvation, if it occurs during periods of stress and medication, may result in life-threatening ketoacidosis, even among non-diabetic women and non-pregnant patients. Awareness of this condition may facilitate prompt recognition and proactive treatment for dietary and stress control.

## Introduction

Ketoacidosis is defined as metabolic acidosis secondary to accumulation of ketone bodies. Diabetic ketoacidosis is the most common cause of ketoacidosis, followed by alcoholic ketoacidosis and rarely starvation ketoacidosis. Starvation-induced ketoacidosis in non-diabetic and non-pregnant, otherwise healthy patients is not common. In a normal healthy individual, short-term starving will only result in mild ketosis. Nonetheless, the effects of ketosis can become more severe if there is a relatively stress and insulin resistance, such as in pregnant or lactating woman or in very young individual such as neonates [1-5]. We report a unique case of severe starvation-induced ketoacidosis in a non-diabetic and non-pregnant patient with a history of multiple recurrent pancreatitis. To our knowledge, the presenting case was novel as no case reports or case series have been reported in these groups of patients.

## Case presentation

A 37-year-old African American female with a past medical history of controlled asthma (no recent exacerbation or corticosteroid use) and multiple episodes of recurrent pancreatitis that is not related to diabetes (last episode was one year prior to admission) presented to the emergency department with abdominal pain, nausea and vomiting over two days. The patient described the lower abdominal pain as sharp and intermittent in nature, rated as 10/10 in intensity, radiating to the back, with no worsening or relieving factors and associated with multiple bouts of nausea and vomiting. The patient reported starving for two days prior to admission, as she had not been able to take in foods and worried that food would worsen the abdominal pain. Otherwise, the patient denied fever, chills, rigors, changes in bowel movement and urinary symptoms. The patient also denied vaginal discharge, and reported no history of sexually transmitted diseases or oral contraceptives and was not sexually active. The patient’s last menstrual period was a few days prior to admission. She also denied any recent sick contact or travel. The patient was not on any medications and supplements such as acetaminophen, salicylates, isoniazid or iron. The patient also denied the use of tobacco, alcohol and illicit drugs.

**Figure 1:**
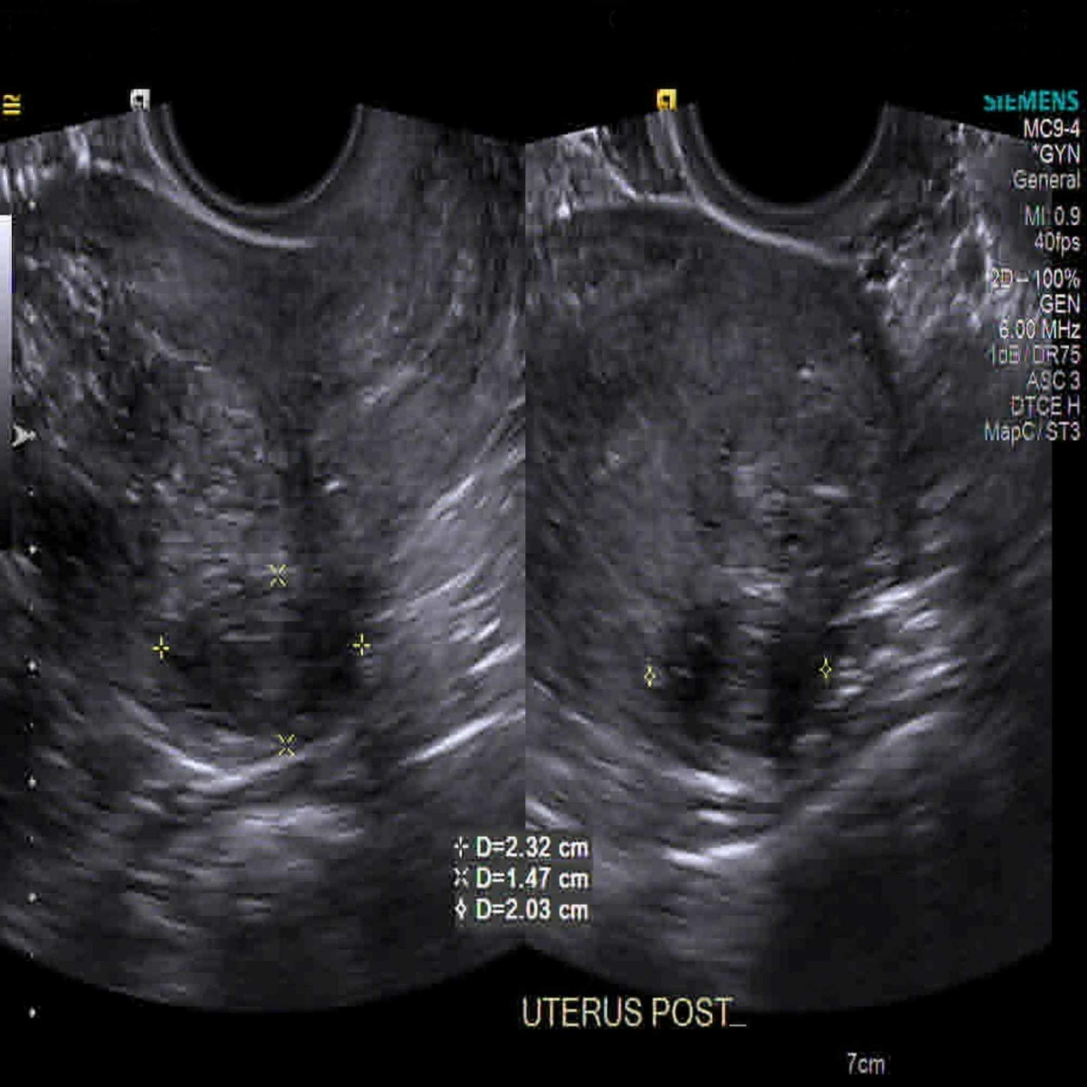
Partially subserosal fibroid along the left posterior fundus, measuring 2.3 x 1.5 x 2 cm

**Figure 2:**
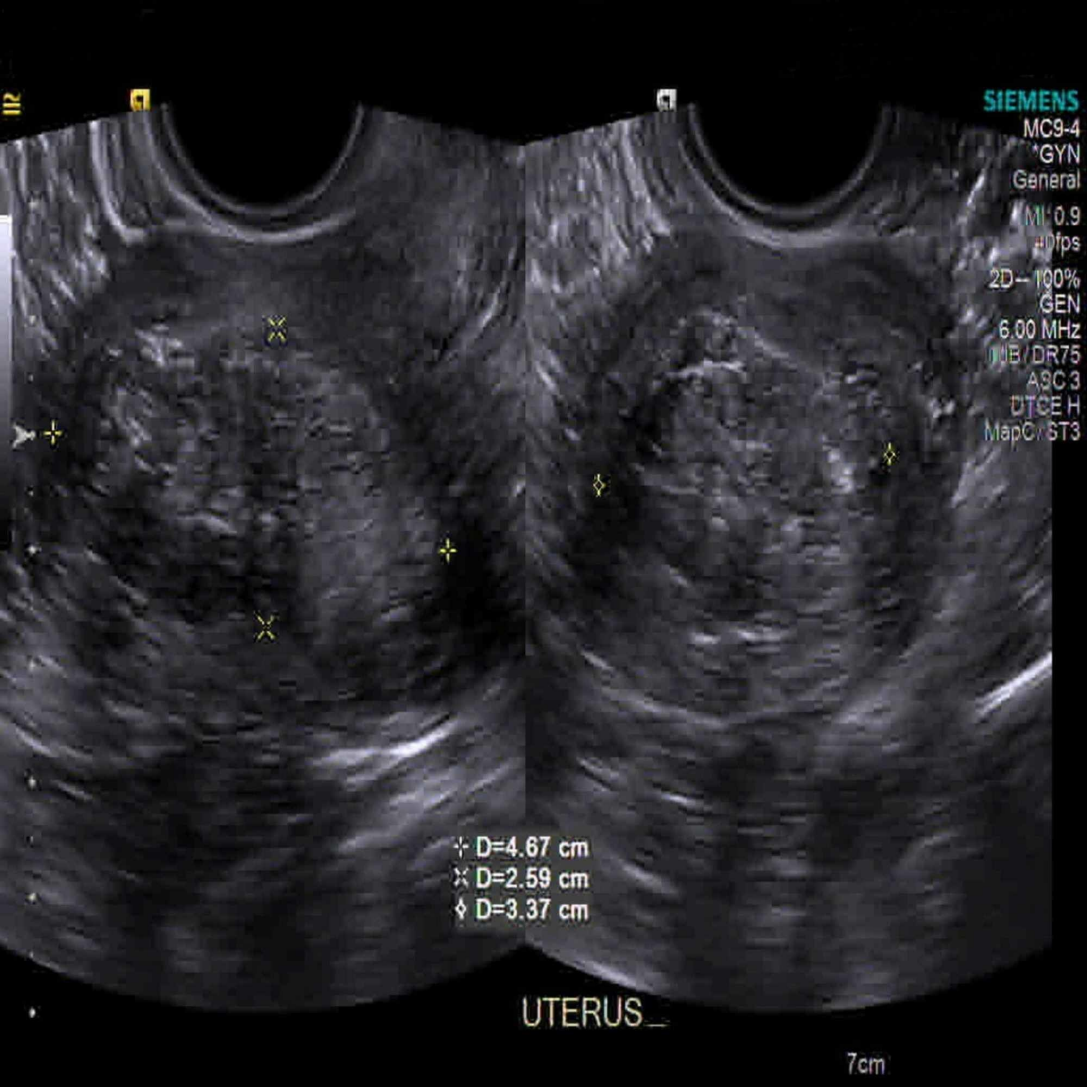
Intramural fibroid along the left posterior fundus, measuring 4.7 x 2.6 x 3.4 cm

In the emergency room, the patient started to complain of shortness of breath, tachypnea and acute respiratory distress. Initial vitals showed a blood pressure of 152/104 mmHg, a heart rate of 142 bpm, a respiratory rate of 24 bpm and a saturation rate of 98% on 2 L/min of oxygen. Abdominal examination showed soft, non-distended, diffuse tenderness over the lower abdomen, no hepatosplenomegaly and normal bowel sounds. Lung and cardiovascular examinations were otherwise normal. Complete metabolic profile showed sodium 132 mmoL/L, potassium 6.4 mmoL/L, bicarb 5 mmoL/L, chloride of 100 mmoL/L, corrected anion gap 29 mmoL/L, lipase was 97 U/L and HbA1c 4.4%. Arterial blood gas showed pH 6.88, pCO_2_ 12 mmHg, pO_2_ 162 mmHg and HCO_3_ 2.7 mmHg. Delta (delta) gap was 0.9, and the patient was experiencing mixed anion gap metabolic acidosis. Urine anion gap was 31.7 mmol/L, with urine pH of 5.5, 2+ protein, no glycosuria and no urine casts observed on urine microscopy. Serum glucose was normal (128 mg/dL) and 3+ ketonuria were present. Lactic acid was 1.7 mmol/L with no uremia (blood urea nitrogen was 6 mg/dL). Lipase was 73 U/L (within normal range). Salicylate acid, acetaminophen and ethanol level were normal. The patient’s beta-hydroxybutyrate level was more than 4.5 and 3+ ketonuria, suggestive of ketoacidosis as the cause of metabolic acidosis. The workup for methanol, ethylene glycol, isopropanol and paraldehyde was negative; however, the patient had an elevated osmolar gap, with a gap of >20 which was thought to be from the ketoacidosis. Chest X-ray was unremarkable. EKG showed sinus tachycardia (with heart rate of 130 bpm), normal axis and no ST-T wave abnormalities. CTs of the abdomen and pelvis did not show any signs of acute pancreatitis, pancreatic atrophy or peripancreatic fluids. There was a large uterine fibroid noted at the endometrial cavity. A transvaginal ultrasound showed two discrete fibroids measuring 2.3 x 1.5 x 2 cm and 4.7 x 2.6 x 3.4 cm on the subserosa (Figure 1) and intramural (Figure 2) of the endometrium, respectively. The lower abdominal pain may have been related to the uterine fibroids, as she had negative pregnancy test and no previous gynecological problems. 

We diagnosed the patient with starvation ketoacidosis due to vomiting, two days of starvation and the absence of other causes of high anion gap metabolic acidosis. Patient was given six ampoules of sodium bicarbonate at the emergency department and was then sent to intensive care unit (ICU). The patient received supplemental oxygen, dextrose and a bicarbonate drip in the ICU. The patient developed refeeding symptoms with hypokalemia, hypomagnesemia and hypophosphatemia. Electrolytes were replenished, and patient’s anion gap was improved and resolved with dextrose and fluid infusion.

## Discussion

Ketoacidosis is defined as metabolic acidosis secondary to accumulation of ketone bodies, namely acetone, acetoacetate and beta-hydroxybutyrate. Of these three known ketone bodies, beta-hydroxybutyric acid is a hydroxy acid (as its name implies), acetone is a pure ketone and acetoacetic acid is a true chemical ketoacid. The generation of ketone bodies in the liver is typically stimulated when there is a low insulin/glucagon ratio. Low insulin levels due to either absolute or relative hypoglycemia (catabolic state) will activate hormone-sensitive lipases, leading to the fatty acid generation from lipolysis of adipose tissues and ultimately transported to liver through bloodstream for the production of ketone bodies via beta-oxidation. The hepatic generation of ketone bodies is a normal physiological response to fasting, and it produces an alternative energy source for the brain and other vital organs [1,6-9].

Mild ketosis generally develops after 12 to 14 hours of fasting. If fasting continues, the ketoacid concentration in the plasma will continue to rise, peaking after 20 to 30 days at a concentration of 8 to 10 mmol/L, at which level the hepatic ketone body synthesis equilibrates with brain and other essential organ tissues’ utilization of those ketone bodies, with beta-hydroxybutyrate being the major accumulating ketone body [1.8]. 

There are at least three stabilization mechanisms inside the body to prevent severe ketoacidosis in patients that are fasting, namely stimulation of insulin release, increased sensitivity of adipose tissue to insulin's inhibitory effect on fatty acid release and direct inhibition of lipolysis by the ketone bodies [10]. Additionally, there will also be an increased rate of central nervous system ketoacid uptake and peripheral tissue ketone utilization. These typically decrease the ketoacid in the body and prevent the development of severe ketoacidosis [11,12].

Our case is unique as the patient was otherwise healthy, non-diabetic, non-pregnant and non-lactating but presented with severe starvation ketoacidosis. We postulate that our patient may have had a defect in the breaking mechanisms. Our theory indicates that due to the multiple bouts of acute pancreatitis in the past, the patient’s pancreas was unable to release insulin appropriately in response to severe ketoacidosis. It is also interesting to note that the patient developed severe starvation ketoacidosis with just two days of starving. It usually takes much longer for this to happen. In the literature, there are case reports discussing the association of low carbohydrate diet with ketosis. Our patient might have had a low carbohydrate diet lately, along with acute stressful conditions such as vomiting and starving that led to the development of severe starvation-induced ketoacidosis 

## Conclusions

Short-term starvation, if it occurs during periods of stress, may result in life-threatening ketoacidosis, even among non-diabetic women and non-pregnant patients. Awareness of this condition may facilitate prompt recognition and proactive treatment for dietary and stress control, especially for malnourished patients; emergent interventions may also improve.
